# Effect of High Intensity Interval Training Compared to Continuous Training on Cognitive Performance in Young Healthy Adults: A Pilot Study

**DOI:** 10.3390/brainsci10020081

**Published:** 2020-02-04

**Authors:** Said Mekari, Meghan Earle, Ricardo Martins, Sara Drisdelle, Melanie Killen, Vicky Bouffard-Levasseur, Olivier Dupuy

**Affiliations:** 1 Department of Kinesiology, Acadia University, Wolfville, NS B4P 2R6, Canada; mearle@kinduct.com (M.E.); rs16eg@brocku.ca (R.M.); sajdrisdelle@uwaterloo.ca (S.D.); melanie.n.killen@gmail.com (M.K.); 2 Sector of Education and Kinesiology, University of Moncton, Edmundston Campus, Edmundston, NB E3V 2S8, Canada; vicky.bouffard-levasseur@umoncton.ca; 3 Laboratory MOVE (EA 6314), Faculty of Sport Sciences, University of Poitiers, 17000 Poitiers, France; olivier.dupuy@univ-poitiers.fr

**Keywords:** exercise physiology, cognition, high intensity interval training, moderate intensity continuous exercise, exercise training

## Abstract

To improve cognitive function, moving the body is strongly recommended; however, evidence regarding the proper training modality is still lacking. The purpose of this study was therefore to assess the effects of high intensity interval training (HIIT) compared to moderate intensity continuous exercise (MICE), representing the same total training load, on improving cognitive function in healthy adults. It was hypothesized that after 6 weeks (3 days/week) of stationary bike training, HIIT would improve executive functions more than MICE. Twenty-five participants exercised three times a week for 6 weeks after randomization to the HIIT or MICE training groups. Target intensity was 60% of peak power output (PPO) in the MICE group and 100% PPO in the HIIT group. After training, PPO significantly increased in both the HIIT and MICE groups (9% and 15%, *p* < 0.01). HIIT was mainly associated with a greater improvement in overall reaction time in the executive components of the computerized Stroop task (980.43 ± 135.27 ms vs. 860.04 ± 75.63 ms, *p* < 0.01) and the trail making test (42.35 ± 14.86 s vs. 30.35 ± 4.13 s, *p* < 0.01). T exercise protocol was clearly an important factor in improving executive functions in young adults.

## 1. Introduction

The positive effects of physical activity (movements carried out by the muscles that require energy) and exercise (planned, structured and intentional movement) [1] on brain function and its metabolism are well known. There is extensive research showing that regular physical activity and exercise can improve cardiorespiratory function and body composition while lowering the risk of chronic disease and mortality [2,3]. It is also well documented that age-related cognitive decline is heterogeneous and several factors modulate the impact of aging on cognition [4]. Exercise intervention studies have supported this and demonstrated that low-intensity aerobic training significantly improves cognitive functions [5]. Interestingly, most studies have documented larger positive impacts of exercise in tasks that involve the prefrontal cortex of the brain [6]. This brain region is involved in many cognitive processes including attention, decision-making, executive function, and working memory [5]. The relationship between aerobic fitness level and attentional control has also been confirmed in a very well-cited meta-analysis of cognitive improvement through aerobic training [7]. Although many aspects of cognitive performance improved significantly after an aerobic training regimen that enhanced cardiorespiratory function, the largest improvement was observed in tasks that implied heavily on executive functions.

The mechanisms related to the beneficial effect of aerobic fitness on brain regions involved with cognitive function are now clearer. Recent findings suggest that an improvement of cardiorespiratory fitness (i.e., $\dot{V}O_{2}$ max) can induce changes to cellular and molecular pathways that likely initiate changes to the macroscopic properties of the brain and behavior, which in turn can influence cognitive functions in the prefrontal cortex of the brain [8].

Evidence from animal studies also suggests that increasing physical activity can enhance synaptogenesis (i.e., formation of new neuronal synapses), and neurogenesis (i.e., generation of new neurons) via increased production of brain-derived neurotrophic factor, in addition to vascular plasticity [9,10]. Hypotheses proposed to account for the relationship between aerobic fitness and cognition include corresponding increases in vascularization of brain tissue [11]. Several authors reported that fitter subjects displayed better cerebral oxygenation during cognitive tasks, which was associated with better vascularization [12,13].

There is a paucity of information available to determine what type of aerobic exercise training program is most optimal for improving cognitive functions. Several models suggest that replacing aerobic exercise training performed at moderate-intensity (i.e., moderate-intensity continuous exercise (MICE)) with high-intensity intermittent training (HIIT) may be considerably more effective at improving cardiovascular [14,15] and cognitive health [16]. Although first described in the 1950s as a mode of cardiac rehabilitation by the German cardiologist Hans Reindell [17], HIIT has largely been used by elite athletes for aerobic training purposes [18,19,20]. HIIT consists of alternating periods of intensive aerobic exercise with periods of recovery. It is well established that HIIT training induces a greater increase in cardiac output and stroke volume than MICE training [21,22,23,24,25]. Most importantly, two recent studies demonstrated that HIIT training is both safe and well-tolerated, without evidence of myocardial damage, significant arrhythmias or left ventricular dysfunction [26,27].

Based on previous evidence in which HIIT appeared to be an efficient training method for improving cardiovascular health, a growing research interest concerning the link between intensity training and cognitive function has appeared. A relationship between exercise intensity training and cognitive function seems to be emerging. Using a questionnaire of physical activity level, Van Gelder et al. [28] reported that older adults who exercised at the lowest intensity were more likely to develop dementia 10 years later compared with those who exercised at higher intensity. In this line, Angeraven et al. [29], using the same methodology as a previous report, found that the average intensity of weekly physical activities of middle aged and older adults was positively associated with cognitive performance. More recently, using actigraphy, Brown et al. [30] indicated that intensity rather than quantity of physical activity might be more important in the association between physical activity and cognitive function. Although these results are encouraging, there is no clear evidence that HIIT has a superior effect on cognitive function compared to MICE [16]. Recently, original studies have provided more responses with null [31,32] or positive results [33,34,35,36,37] in animal and human studies. Among the studies that achieved positive results, they only compared HIIT to active controls, and as such, the effects of exercise intensity per se were not examined. Only Kovacevic et al. [34] found that HIIT had a greater impact on cognitive function than MICE in older adults. In younger adults, the evidence that HIIT is the optimal strategy to improve cognitive performance is unclear and the only data available is contradictory.

The aim of this study was to compare a HIIT and MICE program on cognition in young adults and test the hypothesis that HIIT may be a better strategy to improve cognitive function. Based on the evidence that HIIT has a superior impact on cardiorespiratory health, we put forward the hypothesis that cognition was most affected by this form of exercise and executive function was most sensible to this program.

## 2. Methods

### 2.1. Participants

In this study, 25 young adults (18 females and 7 males) gave their written informed consent to participate in the study. Their parameters (mean ± SD) were: age (32 ± 8 years), height (1.69 ± 0.02 m), body mass (76 ± 17 kg), body mass index (BMI) (27 ± 6 kg m^−2^), peak power output (195 ± 44 W) and $\dot{V}O_{2}$ peak (37 ± 8 mL min^−1^ kg^−1^). All participants were healthy and had normal-to-corrected vision. None of the participants had a history of neurological or psychiatric disorder, color blindness, surgery with general anesthesia in the past 6 months, involuntary tremors, epilepsy or drug/alcohol problems. The protocol was reviewed and approved by the Institutional Research Ethics Board in the Health Sciences of Acadia University (REB 15-09) and was conducted in accordance with the Declaration of Helsinki.

### 2.2. Experimental Design

On the first and last visit, participants underwent a complete physical and cognitive evaluation that included measurement of height, weight, cognitive functions, and a maximal continuous graded exercise test. In order to minimize known confounding influences during exercise testing, participants were asked to refrain from consuming caffeine or smoking within 2 h and drinking alcohol within 6 h of any testing, consistent with the exercise testing guidelines from the Canadian Society of Exercise Physiology (CSEP). Participants were also asked to refrain from heavy exercise 24 h prior to any testing. During the six subsequent weeks, participants were randomly assigned to one of the two experimental protocols: MICE (*n* = 13) or HIIT (*n* = 12) on a stationary bicycle. We used a stratified randomization procedure to ensure that both groups were balanced at baseline for gender and fitness levels. All trainings were under the supervision of an exercise physiologist. Cycling position, which is known to affect energy expenditure, was standardized by adopting a top bar position. Saddle height was adjusted according to the participant’s inseam leg length.

### 2.3. Maximal Continuous Graded Exercise Test

This test was performed on cycle ergometer (Lode B.V., Groningen, Netherlands). Initial workload was set at 1 W/kg body mass, for example 75 W for an individual with a weight of 75 kg. The workload was increased by 15 W every minute until voluntary exhaustion. Strong verbal encouragement was given throughout the test. The power of the last completed stage was considered as the peak power output (PPO, measured in W). Oxygen uptake ($\dot{V}O_{2}$ max, in ml min^−1^ kg^−1^) was determined continuously on a 30 s basis using an automated cardiopulmonary exercise system (Parvo Medics TrueOne 2400, UT, USA). Gas analyzers were calibrated before each test using a gas mixture of known concentration (15% O_2_ and 5% CO_2_). The turbine was calibrated before each test using a 3-L syringe at several flow rates. The highest $\dot{V}O_{2}$ max over a 30 s period during the test was considered as the peak oxygen uptake ($\dot{V}O_{2}$ peak, in ml min^−1^ kg^−1^).

### 2.4. Cognitive Testing

#### 2.4.1. Computerized Modified Stroop Task

The computerized modified Stroop task was based on the modified Stroop color test [2]. This test includes four conditions. In the first condition (Congruent), the participant had to read 1 of 4 possible words appearing on the screen; “RED”, “BLUE”, “YELLOW” or “GREEN”. These words were written in the same colour as their meaning. The answers were mapped to the letters “u”, “i”, “o” and “p” on a keyboard, which participants used to give their answers with the right hand. The mapping remained the same throughout the task. The order was “index finger—red”, “middle finger—green”, “ring finger—blue”, and “little finger—yellow”. The second block consisted in a Denomination condition, where participants had to identify the colour of unrelated words, which were “BUT”, “FOR”, “WHEN”, and “THAN”. The third block consisted in a classic Interference task, which requires naming the colour of a colour-word, the meaning of the word being incongruent with the colour itself (e.g., the word BLUE written in green). In these first three blocks, a fixation cross appeared for 500 ms, followed by the word for 3000 ms. The fourth block consisted in a Switching task, which was identical to the Interference task, except that for 25% of the trials a square appeared instead of the fixation cross, and participants were asked to read the colour-word, instead of naming its colour. The reading trials appeared randomly throughout the block. Each of the four blocks contained 60 trials and the screen was blank between the trials. Before each condition, participants completed practice trials; 12 for the Congruent condition, 5 for the Denomination condition, 12 for the Interference condition, and 20 for the Switching condition. During practice and experimental trials a visual feedback (“Error”) was given for incorrect responses only. Reaction times and errors were recorded.

#### 2.4.2. Trail Making Test

All participants completed the trail making test part A prior to completing the trail making test part B. The trail making test part A (Trail A) was used to measure an individuals’ processing speed. Participants were encouraged to correct their errors and this was included in the total time to complete. The speed at which all the numbers were connected was measured in seconds (s). Part B (Trail B) was used to measure cognitive flexibility or switching ability. In this portion of the test, participants were given the same instructions as Part A but had to alternate between numbers in ascending order and letters in alphabetical order (1-A-2-B-3-C, etc.). The time to complete Part B was also measured in seconds. Prior to the standard administration of this test, participants were given a short practice of each test [38].

### 2.5. Training

For both training protocols, all sessions were supervised and were conducted 3 days per week (Mondays, Wednesdays and Fridays) for 6-weeks. Eighty percent of the 18 sessions had to be completed to be eligible for post-testing. Training intensities for both groups were determined by percentages of their PPO found during the $\dot{V}O_{2}$ max test. Resistance was adjusted to maintain a cycling cadence between 50 and 70 revolutions per minute (rpm). For a more precise load monitoring and exercise prescription, we decided to use an external load measurement of intensity (%PPO) instead of an internal load measurement of intensity (% maximal heart rate). Training methodology was based on a recent paper from O’Brien et al. [39].

### 2.6. Moderate-Intensity Continuous Exercise

The MICE protocol was based on the recommendations of the Canadian Society of Exercise Physiology (CSEP), suggesting that individuals should accumulate at least 150 min of moderate to vigorous physical activity per week. We opted for continuous cycling at 60% PPO for 34 min. Duration was adjusted to match the total calorie expenditure of the HIIT (MICE = 122 kJ vs. HIIT = 120 kJ, for an individual with a PPO of 100 W). To adjust for predicted fitness improvements, the time was increased to 39 min for the remaining four weeks, and during the last two weeks, the intensity was increased by 15 Watts. The participants tapered in the last two training sessions by cycling at the same intensity (initial PPO + 15 Watts) for 30 min. We chose to include the warm up and recovery in the exercise session. The warm-up and cool-down involved 5 min of cycling at 25% PPO.

### 2.7. High-Intensity Intermittent Training

**Figure 1:**
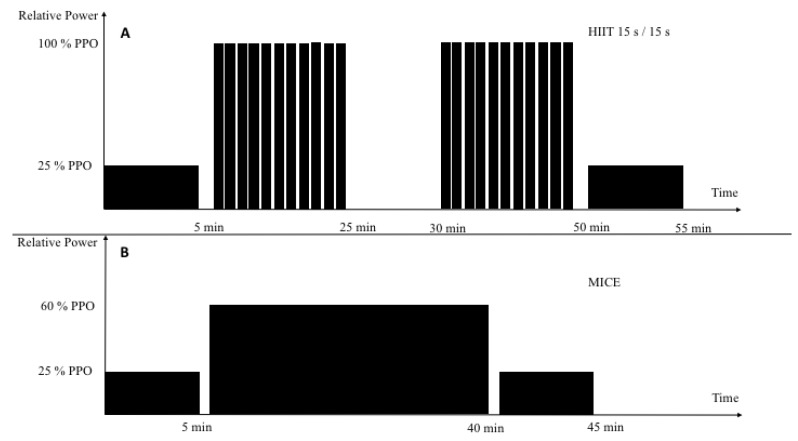
Schematic illustration of our two training modalities. Each training session was preceded by a 5-min standardized warm-up followed by a 5 min passive recovery. HIIT training (**A**) was 15 s at 100% of PPO and 15 s passive recovery (2 × 20-min). MICE training (**B**) was a 34-min exercise at 60% of PPO. Note: *PPO*, peak power output; *HIIT*, high intensity interval training; *MICE*, moderate intensity continuous exercise.

The HIIT session was based on previous studies that compared the time to exhaustion, participant preference, and time spent near $\dot{V}O_{2}$ max of various interval protocols [40]. The group performed 15 s intervals at 100% PPO with 15 s of passive recovery between. The intervals were done for two sets of 20 min (40 min total), with five minutes of passive recovery in between. This was performed for the first two weeks and then increased to a total of 45 min for the remaining four weeks. During the last two weeks, the intensity was increased by 15 Watts. The last two training sessions (tapering sessions) were performed at the same intensity for a total of 35 min. The warm up and recovery were equivalent to the MICE protocol of 5 min each at 25% PPO. Training procedures for both the MICE and HIIT are presented in Figure 1.

## 3. Statistical Analysis

Standard statistical methods were used for the calculation of means and standard deviations. Normal Gaussian distribution of the data was verified by the Shapiro–Wilk test and homoscedascticity by a modified Levene Test. The compound symmetry, or sphericity, was checked by the Mauchley test. When the assumption of sphericity was not met, the significance of F-ratios was adjusted according to the Greenhouse–Geisser procedure when the epsilon correction factor was <0.75, or according to the Huyn–Feld procedure when the epsilon correction factor was >0.75. On each physiological measure, an analysis of variance (ANOVA; time × training group) was conducted. For the trail making test, a 2 × 2 ANOVA was conducted to examine (time × training group). For the Stroop test, a 2 × 2 ANOVA was conducted to test (time × training group). All post-hoc tests were Bonferroni corrected for multiple comparisons. The magnitude of the difference between fitness levels was assessed by the Hedges’ g (g), as presented elsewhere [41]. The magnitude of the difference was considered either small (0.2 < ES < 0.5), moderate (0.5 < ES < 0.8), or large (ES > 0.8). The significance level was set at *p* < 0.05 for all analyses.

## 4. Results

### 4.1. Maximal Continuous Graded Exercise Test

At baseline, there was no significant difference in maximal aerobic power or anthropometrics measured between both groups (Table 1). After the 6 week training protocol, $\dot{V}O_{2}$ max increased significantly for both the MICE group and the HIIT group (main effect of time F(1,22) = 15.9). The MICE group saw a maximal aerobic power increase from 180 ± 41 to 213 ± 43 W (*p* < 0.05) and the HIIT group also saw an increase from 207 ± 44 to 217 ± 42 W (*p* < 0.05).

### 4.2. Cognitive Test

#### 4.2.1. Stroop Task

Concerning the reaction time, the analysis revealed that there was a significant effect of the Stroop Condition (F(1,22) = 61.87; *p* < 0.05), with a longer reaction time (RT) in the more Executive compared to Non-Executive conditions (Stroop 1 < 2 < 3 < 4). In addition, we found that time (F(1,22) = 4.46; *p* < 0.05) and reaction time were shorter after training than before training. Further analysis (pairwise comparisons) also revealed lower RT in the switching task (executive task), which was selectively associated with only HIIT training (*p* < 0.05). Results for RT as a function of group training are presented in Table 2. Concerning accuracy, the ANOVA revealed a main effect of Task (F(3,20) = 8.83; *p* < 0.01) (Stroop 1, 2, 3, are different from 4). No statistical significance was observed between the training protocols.

#### 4.2.2. Trail Making Test

The ANOVA revealed a significant effect of task (F(1,46) = 89.5; *p* < 0.01 (Trail A < Trail B)) and time (F(1,46) = 16.06; *p* < 0.01 (Pre > Post)). In addition, the analysis revealed an interaction of Task x time (F(1,46) = 7.83; *p* < 0.001). The post-hoc revealed that the performance in Trail A did not change before and after training, whereas the performance in Trail B was improved after training. Interestingly, the ANOVA revealed an interaction of Task × time × Group (F(1,46) = 4.5; *p* < 0.05). A quicker time for completion of the Trail B test was also selectively associated with only the HIIT training (*p* < 0.01). Results for the trail A and B time as a function of group training are presented in Table 2.

**Figure 2:**
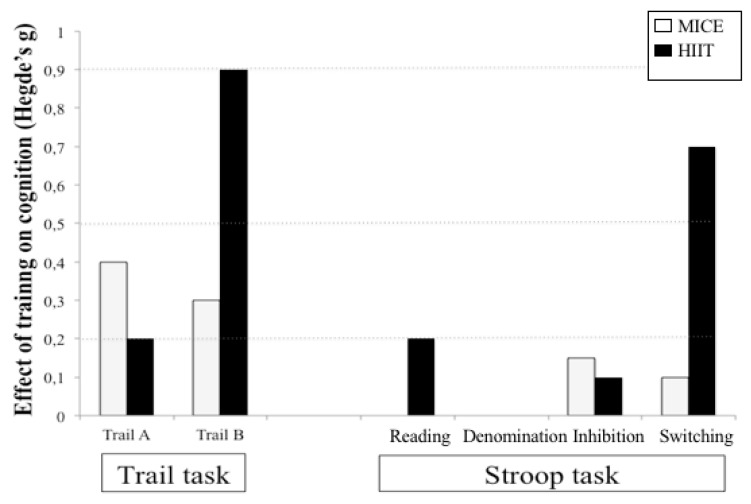
The magnitude of the training effect on cognitive performance for the Trail task and the Stroop task. An increase in effect size corresponds to a decrease in reaction, which means an improvement in cognitive performance.

The magnitude of the training effect (Hedes’s g) for Stroop and Trail are presented in Figure 2.

## 5. Discussion

The aim of this study was to evaluate the impact of HIIT training compared to continuous training on cognitive performance. The results confirm that selectively executive functions are sensitive to HIIT training. Only subjects from the HIIT group enhanced their flexibility performance as measured by the Stroop task and Trail B.

Our first hypothesis that HIIT training would significantly increase $\dot{V}O_{2}$ max was not supported. These findings are in contrast to those by Helgerud et al. [25] who found that there was a significantly greater increase in $\dot{V}O_{2}$ max in HIIT groups compared to MICE groups, where one group used a protocol of 15 s work and 15 s rest, which is similar to the one used in this study. Similarly, a significant increase in $\dot{V}O_{2}$ max in the HIIT group compared to the MICE group was found after 10 weeks of aerobic exercise training (*p* < 0.05) [42]. Our results are in line with those found by Daussin et al. [24] and Kemmler et al. [43], who found a significant increase in $\dot{V}O_{2}$ max for both HIIT and MICE training, but no significant difference between the groups’ respective increases. Both studies still showed a greater increase in aerobic fitness in the HIIT group compared to the MICE group. However, two recent meta-analyses [44,45] confirmed that the superiority of HIIT on $\dot{V}O_{2}$ max is not consistent in young populations and our results are in line with these two recent reports.

Increases in performance on the trail test B were found in the HIIT group and are likely to result from the executive portion of the Stroop task, which also showed an increase in executive function in the HIIT group compared to the MICE group. These findings support our second hypothesis that greater improvements in executive functioning will result from HIIT compared to MICE. The results of our study confirm certain results from the literature in children [35], adolescents [33], young adults [37], or the elderly who are healthy [34] or have had a stroke [36]. In addition, our results on the trail making task are consistent with those reported by Pallesen et al. who reported an effect of intermittent high intensity training only on the Trail B and not on the Trail A [36]. In addition, our results on the Stroop 4 (Flexibilty) are consistent with those reported by Jeaon et al. [33] in adolescents. However, all of these studies did not compare high intensity training with moderate intensity training and often used an active control group. It is therefore difficult to conclude what the effect of intensity was. Our study is the only one that has confirmed the superior effect of HIIT training on executive function in young adults. Because this is the first study known to the researcher that examines the effect that exercise protocol may have on executive function in young adults, it can only be speculated as to the mechanisms that underlie this relationship.

It was previously believed that there was a direct correlation between a high aerobic fitness level and increased levels of executive functioning. Many studies have been able to demonstrate, using aerobic training interventions, that an increase in $\dot{V}O_{2}$ max will elicit improvements in executive functioning. Kramer et al. [6] tested 124 sedentary adults and randomly divided them into aerobic or control groups that trained for 6 months. They found that there was a significant improvement in executive functioning tasks that were correlated with significant improvements in aerobic fitness (5.1% aerobic fitness increase) in the aerobic group only. They concluded that the improvements they found in executive functioning were due to increases in aerobic fitness. Similarly, Colcombe et al. [46] were able to show increases in executive functioning in an aerobic training group that had also improved $\dot{V}O_{2}$ max by 10.2% over the course of a 6-month training period.

In line with the current study, Smiley-Oyen et al. [47] randomly assigned participants to either a moderate-intensity aerobic training group or a toning/control group for 10 months. Aerobic and cognitive tests were performed before and after the 10-month training period with significant improvements in executive function tests found in the aerobic group but not the control group. There was also no significant difference found between $\dot{V}O_{2}$ peak between the two groups at the beginning or the cessation of the training period. The results were similar to those found in this study, in that while there was a significant increase in executive function performance, there was no significant increase between intervention groups. Another study was also able to question the link between aerobic fitness interventions and increased cognitive functions. Madden and colleagues [48] were able to show the opposite effect, after randomly placing older adults into either an aerobic training group, yoga group, or non-intervention group. The participants trained for 32 weeks, with the aerobic group training three times per week for 45 min at a moderate intensity of 70% heart rate reserve (HRR). Participants’ aerobic fitness and cognition were tested, resulting in a significant (*p* < 0.01) increase in $\dot{V}O_{2}$ max in the aerobic group but not the yoga or non-intervention group. While there was a significant increase found in aerobic fitness, there was no significant interaction found between exercise and cognitive function. In addition, a meta-analysis of 37 studies conducted by Eitner et al. [49] did not show evidence to support the relationship between cognition and fitness levels. After analyzing correlational studies, they found that aerobic fitness only accounted for 8% of the cognitive variance. These studies offer further evidence to suggest that there might be more to the relationship between aerobic exercise and cognition than just statistically significant improvements in $\dot{V}O_{2}$ max.

There are two main mechanisms discussed in the literature that may help explain the findings of this study. The first is that exercise may increase the levels of brain-derived neurotrophic factor (BDNF), which could in turn improve cognition. BDNF is a growth factor that encourages neural plasticity and synaptic growth and transmission, and has been shown to enhance cognition due to its up-regulation and role in angiogenesis [9,10,50]. The role of BDNF may be multi-layered, with other enzyme and hormone interactions playing a role on BDNF levels including estrogen, corticosterone, and insulin growth factor-1 (IGF-1) [9]. Even though there are many interactions taking place, exercise has been shown to be the catalyst in BDNF affecting the brain [9]. Indeed, acute exercise is recognized to promote the release of serum BNDF [51] and this release seems to be dependent on exercise intensity [52,53]. For example, Winter and colleagues [54] tested the effect of acute exercise on BDNF serum levels and learning using both HIIT, MICE, and rest interventions. It was found that in the HIIT group, there was an increased level of learning success and this success was related to increased BDNF serum levels (r = 0.38; *p* = 0.05). Exercise also increased BDNF levels in serum or plasma, and HIIT seemed a good alternative to MICE as it produced higher levels of BDNF release [54,55,56,57]. Because levels remained highest after intense training cessation and also improved cognitive function, it is thought that BDNF may be increasingly elicited by HIIT as opposed to MICE. In contrast, a study by Lou et al. [58] on rats showed an intensity-dependent relationship in which mRNA BDNF levels were significantly lower after 4 weeks of high-intensity running compared to low-intensity running (*p* < 0.01). As there is limited research on humans regarding the effect of intensity on BDNF, we are unable to draw a conclusion based on the literature and this study’s results. In saying this, there is evidence suggesting that BDNF plays a key role in cognition, and that BDNF may increase with aerobic exercise training as the chronic production of BDNF seems to mediate improvements in executive function in a long-term intervention [59].

The second mechanism is in regards to increases in cerebral blood flow. Precise mechanisms of the interaction between brain function and exercise are not clearly understood, but cerebral blood flow (CBF) and arterial regulation are thought to play a major role [60]. The cardiovascular hypothesis states that an improvement in cardiovascular function (cardiac output, oxygen transport and metabolism) can lead to improved neurotransmitter function and brain health [61]. Based on the cardiovascular hypothesis, a higher cardiac output typically results in higher cerebral blood flow, implying that the greater cardiovascular adaptation from HIIT should also positively influence cognitive performance. The CBF model is somewhat based on the cardiovascular hypothesis, in that increasing the ability of the heart to pump blood to the body and the brain will thus increase the amount of blood that is being transported to the cerebrum. It has been repeatedly shown that increases in CBF are related to increased cognitive performance and that, as humans age, there is a decline in CBF that is congruent with declines in cognitive function [54]. In a study of 17- to 79-year-old men, Ainslie et al. [60] showed a 17% higher level of CBF in endurance-trained men compared to asymptomatic sedentary men. Recently, Robinson et al. [62] found that the cerebral metabolism was improved only in participants who completed a HIIT training program. These results could explain our cognitive results but further research is needed regarding the relationship between exercise intensity, cerebral metabolism, and cognition.

### Limitations

While there were statistically significant results found in the study, there are still some limitations to consider. The training period length was short, and could have resulted in the lack of significant findings regarding $\dot{V}O_{2}$ max changes. Even though there was an attendance limit of 80%, some participants missed consecutive training sessions, which could have impacted their performance while experiencing detraining effects. In order to control for known physiological influences in exercise (i.e., blood pressure, heart rate, fatigue, hormones, etc.), we attempted to have each participant complete their training sessions early in the morning. Due to prior commitments from the participants, it was difficult for all the participants to complete every session at the same time of day. We also did not keep a record of other activities that the participants were taking part in, such as pre training exercise levels, other workouts or “brain-training” games that could have altered the physical or cognitive results. On other hand, the small sample size could be a limit and these results need to be confirmed by future research. In addition, the authors acknowledged that the cognitive performance in this study was assessed only with the Stroop and trail test and that it is difficult to generalize our findings to cognition in general. A major limitation evident in the study is the lack of measurement regarding potential mechanisms mediating the relationship between exercise protocol and cognition. It would have been beneficial to provide measurements of some of the possible mechanisms, but the positive results of the study imply that a future area of research lies in determining the mechanisms by which exercise influences executive functioning.

## 6. Conclusions

This study adds to research in favor of HIIT over MICE as a more effective way to improve performance of executive function. To our knowledge, this is the first study to investigate the different protocols of aerobic exercise training on executive function in young adults. These findings may be important in the development of programs that are efficient and effective at combating age-related cognitive decline and increasing levels of cognitive functioning in adults. Considering that HIIT is a safe way to improve fitness in older adults and those with chronic disease, further research is warranted.

## Figures and Tables

**Table 1:** Pre- and post-training participant data values for anthropometric and aerobic exercise measures.

	HIIT		MICE	
	PRE	POST	PRE	POST
Age	29 ± 10.3		35 ± 7.4	
Height (m)	1.7 ± 0.1		1.7 ± 0.1	
Gender	9 F, 3 M		9 F, 4 M	
Weight (kg)	71.3 ± 13.0	70.8 ± 13.4	81.3 ± 13.0	82.4 ± 23.1
BMI	24.6 ± 5.0	24.4 ± 5.1	28.8 ± 8.0	29.2 ± 8.2
$\dot{V}O_{2}$ max (mL/kg/min)	39.7 ± 8.7	41 ± 8.4	33.8 ± 8.3	35.9 ± 8.6
MAP (W)	207 ± 44.9	217 ± 42.2 ^a^	180 ± 41.4	213 ± 43.0 ^a^

Note. M, meters; kg, kilograms; BMI, body mass index; MAP, maximal aerobic power; W, Watts; F, Female; M, Male ^a^ Statistically different from PRE, *p* < 0.05.

**Table 2:** Cognitive Responses to Training Intensity Pre and Post Intervention.

	HIIT		MICE	
	PRE	POST	PRE	POST
**Stroop Task (ms)**				
Reading	598.83 ± 99.40	574.15 ±106	604.43 ± 89.12	602.58 ± 95.66
Denomination	646.11 ± 93.67	616.33 ± 99.30	646.11 ± 93.67	644.59 ± 92.10
Inhibition	688.65 ± 95.47	680.94 ± 102.20	721.67 ± 110.07	687.41 ± 86.78
Switching	980.43 ± 135.27	860.04 ± 75.63 ^a^	1008.45 ± 218.76	987.77 ± 188.20
**Trail A (sec)**	16.47 ± 4.76	15.44 ± 3.09	16.64 ± 4.21	16.45 ± 3.42
**Trail B (sec)**	42.35 ± 14.86	30.35 ± 4.13 ^ab^	33.15 ± 7.06	34.13 ± 9.91

Note: Values are expressed in mean ± SD; *ms*, milliseconds; *sec*, seconds; *HIIT*, high intensity interval training; *MICE*, moderate intensity continuous exercise. Compared with PRE HIIT training: ^a^ *p* < 0.05, Compared to MICE training ^b^ *p* < 0.02.

## References

[1] Budde H., Schwarz R., Velasques B., Ribeiro P., Holzweg M., Machado S., Brazaitis M., Staack F. (2016). Wegner M8.The need for differentiating between exercise, physical activity, and training. Autoimmun. Rev..

[2] Kodama S., Saito K., Tanaka S., Maki M., Yachi Y., Asumi M., Sugawara A., Totsuka K., Shimano H., Ohashi Y. (2009). Cardiorespiratory fitness as a quantitative predictor of all-cause mortality and cardiovascular events in healthy men and women: A meta-analysis. JAMA.

[3] Tremblay M.S., Warburton D.E., Janssen I., Paterson D.H., Latimer A.E., Rhodes R.E., Kho M.E., Hicks A., Leblanc A.G., Zehr L. (2011). New Canadian physical activity guidelines. Appl. Physiol. Nutr. Metab..

[4] Bherer L., Erickson K.I., Liu-Ambrose T. (2013). A review of the effects of physical activity and exercise on cognitive and brain functions in older adults. J. Aging Res..

[5] Kramer A.F., Erickson K.I., Colcombe S.J. (2006). Exercise, cognition, and the aging brain. J. Appl. Physiol. (1985).

[6] Kramer A.F., Hahn S., Cohen N.J., Banich M.T., McAuley E., Harrison C.R., Chason J., Vakil E., Bardell L., Boileau R.A. (1999). Ageing, fitness and neurocognitive function. Nature.

[7] Colcombe S., Kramer A.F. (2003). Fitness effects on the cognitive function of older adults: A meta-analytic study. Psychol. Sci..

[8] Stillman C.M., Cohen J., Lehman M.E., Erickson K.I. (2016). Mediators of Physical Activity on Neurocognitive Function: A Review at Multiple Levels of Analysis. Front. Hum. Neurosci..

[9] Cotman C.W., Berchtold N.C. (2002). Exercise: A behavioral intervention to enhance brain health and plasticity. Trends Neurosci..

[10] Cotman C.W., Berchtold N.C., Christie L.A. (2007). Exercise builds brain health: Key roles of growth factor cascades and inflammation. Trends Neurosci..

[11] Bullitt E., Rahman F.N., Smith J.K., Kim E., Zeng D., Katz L.M., Marks B.L. (2009). The effect of exercise on the cerebral vasculature of healthy aged subjects as visualized by MR angiography. AJNR Am. J. Neuroradiol..

[12] Dupuy O., Gauthier C.J., Fraser S.A., Desjardins-Crepeau L., Desjardins M., Mekary S., Lesage F., Hoge R.D., Pouliot P., Bherer L. (2015). Higher levels of cardiovascular fitness are associated with better executive function and prefrontal oxygenation in younger and older women. Front. Hum. Neurosci..

[13] Mekari S., Dupuy O., Martins R., Evans K., Kimmerly D.S., Fraser S., Neyedli H.F. (2019). The effects of cardiorespiratory fitness on executive function and prefrontal oxygenation in older adults. Geroscience.

[14] Cao M., Quan M., Zhuang J. (2019). Effect of High-Intensity Interval Training versus Moderate-Intensity Continuous Training on Cardiorespiratory Fitness in Children and Adolescents: A Meta-Analysis. Int. J. Environ. Res. Public Health.

[15] Ito S. (2019). High-intensity interval training for health benefits and care of cardiac diseases—The key to an efficient exercise protocol. World J. Cardiol..

[16] Lucas S.J., Cotter J.D., Brassard P., Bailey D.M. (2015). High-intensity interval exercise and cerebrovascular health: Curiosity, cause, and consequence. J. Cereb. Blood Flow Metab..

[17] Reindell H., Roskamm H. (1959). Ein Beitrag zu den physiologischen Grundlagen des Intervall training unter besonderer Berück- sichtigung des Kreilaufes. Schweiz Z Sportmed.

[18] Billat V.L. (2001). Interval training for performance: A scientific and empirical practice. Special recommendations for middle and long distance running. Part II: Anaerobic interval training. Sports Med..

[19] Billat V.L. (2001). Interval training for performance: A scientific and empirical practice. Special recommendations for middle and long distance running. Part I: Aerobic interval training. Sports Med..

[20] Daniels J.T., Scardina N. (1984). Interval training and performance. Sports Med..

[21] Gaitanos G.C., Williams C., Boobis L.H., Brooks S. (1993). Human muscle metabolism during intermittent maximal exercise. J. Appl. Physiol..

[22] Laursen P.B., Jenkins D.G. (2002). The scientific basis for high intensity interval training: Optimising training programmes and maximising performance in highly trained endurance athletes. Sports Med..

[23] Daussin F.N., Ponsot E., Dufour S.P., Lonsdorfer-Wolf E., Doutreleau S., Geny B., Piquard F., Richard R. (2007). Improvement of VO2max by cardiac output and oxygen extraction adaptation during intermittent versus continuous endurance training. Eur. J. Appl. Physiol..

[24] Daussin F.N., Zoll J., Dufour S.P., Ponsot E., Lonsdorfer-Wolf E., Doutreleau S., Mettauer B., Piquard F., Geny B., Richard R. (2008). Effect of interval versus continuous training on cardiorespiratory and mitochondrial functions: Relationship to aerobic performance improvements in sedentary. Am. J. Physiol..

[25] Helgerud J., Hoydal K., Wang E., Karlsen T., Berg P., Bjerkaas M., Simonsen T., Helgesen C., Hjorth N., Bach R. (2007). Aerobic high-intensity intervals improve VO2max more than moderate training. Med. Sci. Sports Exerc..

[26] Juneau M., Roy N., Nigam A., Tardif J.C., Larivee L. (2009). Exercise above the ischemic threshold and serum markers of myocardial injury. Can. J. Cardiol..

[27] Noël M., Jobin J., Marcoux A., Poirier P., Dagenais G., Bogaty P. (2007). Can prolonged exercise-induced myocardial ischaemia be innocuous?. Eur. Heart J..

[28] van Gelder B.M., Tijhuis M.A., Kalmijn S., Giampaoli S., Nissinen A., Kromhout D. (2004). Physical activity in relation to cognitive decline in elderly men: The FINE Study. Neurology.

[29] Angevaren M., Vanhees L., Wendel-Vos W., Verhaar H.J., Aufdemkampe G., Aleman A., Verschuren W.M. (2007). Intensity, but not duration, of physical activities is related to cognitive function. Eur. J. Cardiovasc. Prev. Rehabil..

[30] Brown B.M., Peiffer J.J., Sohrabi H.R., Mondal A., Gupta V.B., Rainey-Smith S.R., Taddei K., Burnham S., Ellis K.A., Szoeke C. (2012). Intense physical activity is associated with cognitive performance in the elderly. Transl. Psychiatry.

[31] Freitas D.A., Rocha-Vieira E., De Sousa R.A.L., Soares B.A., Rocha-Gomes A., Chaves Garcia B.C., Cassilhas R.C., Mendonca V.A., Camargos A.C.R., De Gregorio J.A.M. (2019). High-intensity interval training improves cerebellar antioxidant capacity without affecting cognitive functions in rats. Behav. Brain Res..

[32] Nicolini C., Toepp S., Harasym D., Michalski B., Fahnestock M., Gibala M.J., Nelson A.J. (2019). No changes in corticospinal excitability, biochemical markers, and working memory after 6 weeks of high-intensity interval training in sedentary males. Physiol. Rep..

[33] Jeon Y.K., Ha C.H. (2017). The effect of exercise intensity on brain derived neurotrophic factor and memory in adolescents. Environ. Health Prev. Med..

[34] Kovacevic A., Fenesi B., Paolucci E., Heisz J.J. (2019). The effects of aerobic exercise intensity on memory in older adults. Appl. Physiol. Nutr. Metab..

[35] Moreau D., Kirk I.J., Waldie K.E. (2017). High-intensity training enhances executive function in children in a randomized, placebo-controlled trial. Elife.

[36] Pallesen H., Bjerk M., Pedersen A.R., Nielsen J.F., Evald L. (2019). The Effects of High-Intensity Aerobic Exercise on Cognitive Performance After Stroke: A Pilot Randomised Controlled Trial. J. Cent. Nerv. Syst. Dis..

[37] Venckunas T., Snieckus A., Trinkunas E., Baranauskiene N., Solianik R., Juodsnukis A., Streckis V., Kamandulis S. (2016). Interval Running Training Improves Cognitive Flexibility and Aerobic Power of Young Healthy Adults. J. Strength Cond. Res..

[38] Bowie C.R., Harvey P.D. (2006). Administration and interpretation of the Trail Making Test. Nat. Protoc..

[39] O’Brien M.W., Johns J.A., Robinson S.A., Bungay A., Mekary S., Kimmerly D.S. (2019). Impact of HIIT, MICT, and Resistance Training on Endothelial Function in Older Adults. Med. Sci. Sports Exerc..

[40] Guiraud T., Juneau M., Nigam A., Gayda M., Meyer P., Mekary S., Paillard F., Bosquet L. (2010). Optimization of high intensity interval exercise in coronary heart disease. Eur. J. Appl. Physiol..

[41] Dupuy O., Lussier M., Fraser S., Bherer L., Audiffren M., Bosquet L. (2014). Effect of overreaching on cognitive performance and related cardiac autonomic control. Scand. J. Med. Sci. Sports.

[42] Rognmo O., Hetland E., Helgerud J., Hoff J., Slordahl S.A. (2004). High intensity aerobic interval exercise is superior to moderate intensity exercise for increasing aerobic capacity in patients with coronary artery disease. Eur. J. Cardiovasc. Prev. Rehabil..

[43] Kemmler W., Scharf M., Lell M., Petrasek C., von Stengel S. (2014). High versus moderate intensity running exercise to impact cardiometabolic risk factors: The randomized controlled RUSH-study. Biomed. Res. Int..

[44] Bacon A.P., Carter R.E., Ogle E.A., Joyner M.J. (2013). VO2max trainability and high intensity interval training in humans: A meta-analysis. PLoS ONE.

[45] Scribbans T.D., Vecsey S., Hankinson P.B., Foster W.S., Gurd B.J. (2016). The Effect of Training Intensity on VO2max in Young Healthy Adults: A Meta-Regression and Meta-Analysis. Int. J. Exerc. Sci..

[46] Colcombe S.J., Kramer A.F., Erickson K.I., Scalf P., McAuley E., Cohen N.J., Webb A., Jerome G.J., Marquez D.X., Elavsky S. (2004). Cardiovascular fitness, cortical plasticity, and aging. Proc. Natl. Acad. Sci. USA.

[47] Smiley-Oyen A.L., Lowry K.A., Francois S.J., Kohut M.L., Ekkekakis P. (2008). Exercise, fitness, and neurocognitive function in older adults: The “selective improvement” and “cardiovascular fitness” hypotheses. Ann. Behav. Med..

[48] Madden D.J., Blumenthal J.A., Allen P.A., Emery C.F. (1989). Improving aerobic capacity in healthy older adults does not necessarily lead to improved cognitive performance. Psychol. Aging.

[49] Etnier J.L., Nowell P.M., Landers D.M., Sibley B.A. (2006). A meta-regression to examine the relationship between aerobic fitness and cognitive performance. Brain Res. Rev..

[50] Best J.R. (2010). Effects of Physical Activity on Children’s Executive Function: Contributions of Experimental Research on Aerobic Exercise. Dev. Rev..

[51] Piepmeier A.T., Etnier J.L. (2015). Brain-derived neurotrophic factor (BDNF) as a potential mechanism of the effects of acute exercise on cognitive performance. J. Sport Health Sci..

[52] Piepmeier A.T., Etnier J.L., Wideman L., Berry N.T., Kincaid Z., Weaver M.A. (2019). A preliminary investigation of acute exercise intensity on memory and BDNF isoform concentrations. Eur. J. Sport Sci..

[53] Schmolesky M.T., Webb D.L., Hansen R.A. (2013). The effects of aerobic exercise intensity and duration on levels of brain-derived neurotrophic factor in healthy men. J. Sports Sci. Med..

[54] Winter B., Breitenstein C., Mooren F.C., Voelker K., Fobker M., Lechtermann A., Krueger K., Fromme A., Korsukewitz C., Floel A. (2007). High impact running improves learning. Neurobiol. Learn. Mem..

[55] Enette L., Vogel T., Fanon J.L., Lang P.O. (2017). Effect of Interval and Continuous Aerobic Training on Basal Serum and Plasma Brain-Derived Neurotrophic Factor Values in Seniors: A Systematic Review of Intervention Studies. Rejuvenation Res..

[56] Jimenez-Maldonado A., Renteria I., Garcia-Suarez P.C., Moncada-Jimenez J., Freire-Royes L.F. (2018). The Impact of High-Intensity Interval Training on Brain Derived Neurotrophic Factor in Brain: A Mini-Review. Front. Neurosci..

[57] Saucedo Marquez C.M., Vanaudenaerde B., Troosters T., Wenderoth N. (2015). High-intensity interval training evokes larger serum BDNF levels compared with intense continuous exercise. J. Appl. Physiol. (1985).

[58] Lou S.J., Liu J.Y., Chang H., Chen P.J. (2008). Hippocampal neurogenesis and gene expression depend on exercise intensity in juvenile rats. Brain Res..

[59] Freitas D.A., Rocha-Vieira E., Soares B.A., Nonato L.F., Fonseca S.R., Martins J.B., Mendonca V.A., Lacerda A.C., Massensini A.R., Poortamns J.R. (2018). High intensity interval training modulates hippocampal oxidative stress, BDNF and inflammatory mediators in rats. Physiol. Behav..

[60] Ainslie P.N., Cotter J.D., George K.P., Lucas S., Murrell C., Shave R., Thomas K.N., Williams M.J., Atkinson G. (2008). Elevation in cerebral blood flow velocity with aerobic fitness throughout healthy human ageing. J. Physiol..

[61] Dustman R.E., Emmerson R.Y., Ruhling R.O., Shearer D.E., Steinhaus L.A., Johnson S.C., Bonekat H.W., Shigeoka J.W. (1990). Age and fitness effects on EEG, ERPs, visual sensitivity, and cognition. Neurobiol. Aging.

[62] Robinson M.M., Lowe V.J., Nair K.S. (2018). Increased Brain Glucose Uptake After 12 Weeks of Aerobic High-Intensity Interval Training in Young and Older Adults. J. Clin. Endocrinol. Metab..

